# Source-only cross-year prediction of perceived AI capability with a theory-anchored, loss-specific two-stage model

**DOI:** 10.3389/fpsyg.2026.1867910

**Published:** 2026-10-21

**Authors:** Dan Wang, Yuanzhi Song

**Affiliations:** School of Business Administration, Liaoning Technical University, Huludao, Liaoning, China

**Keywords:** affective stance, cross-year prediction, human–AI interaction, source-only transfer, theory-guided learning, trust in AI

## Abstract

**Introduction:**

Understanding how developers judge AI's ability to handle complex tasks can help organizations monitor attitudes toward AI at work. Repeated surveys provide an opportunity to study these judgments, but models used across years must combine useful prediction with clear interpretation of the roles of trust and favorability toward AI. Adding work-related information or changing the prediction objective can alter the coefficients used to describe those relationships. This study examines whether these goals can be separated using Theory-Anchored Residual Surface learning (TARS).

**Methods:**

We analyzed repeated cross-sectional data from the official 2024 and 2025 Stack Overflow Developer Surveys (*n* = 22, 757 and 17, 021 employed professional developers). The outcome was respondents' five-category rating of how well AI tools handle complex development tasks. TARS first models its relationship with trust in output accuracy and favorability toward AI, then uses work and demographic information to correct prediction errors while keeping the first-stage coefficients fixed. Model development used 2024 data alone; 2025 outcomes were reserved for evaluation against models trained under the same protocol.

**Results:**

In both surveys, higher trust and favorability were associated with higher perceived capability, with trust showing the stronger association. In the cross-year evaluation, TARS achieved *R*^2^ = 0.445 (95% CI [0.434, 0.456]) and mean absolute error = 0.742. Changing the second-stage tuning objective improved absolute-error prediction while retaining the same first-stage coefficients.

**Discussion:**

These findings show how repeated surveys can support prediction of perceived AI capability while keeping the reported trust-favorability relationship consistent across model adjustments. The approach offers an interpretable way to study professional appraisals of AI as work contexts and prediction priorities change.

## 1 Introduction

Organizations use repeated surveys to study how professionals evaluate AI tools for coding, debugging, information retrieval, and workflow support (Rahwan et al., 2019; Noy and Zhang, 2023; Brynjolfsson et al., 2025). The prospective problem is more demanding. A model developed on one survey wave should remain useful in the next wave, when target-year outcomes are unavailable, without changing the psychological coefficients that readers need to interpret. This source-only cross-year setting matches the information available in annual monitoring before the next outcome collection is complete.

Existing approaches do not provide this separation. A one-stage predictive model estimates the trust and favorability terms together with work and demographic variables. Consequently, changing the context variables can also change the coefficients used for psychological interpretation. Retuning the whole model for a different error measure can change them again. Transductive adaptation methods address a different setting because they use the target feature distribution during fitting. The required estimator must keep the trust–favorability relationship fixed, use context only to correct the remaining prediction error, allow that correction to be tuned for different metrics, and use source-year information alone.

This separation also follows the distinction between explanatory structure and out-of-sample prediction (Shmueli, 2010; Yarkoni and Westfall, 2017). When interpretation is part of the scientific claim, an explicitly interpretable predictive component can be preferable to explaining a black-box model after fitting (Rudin, 2019).

TARS addresses this problem in two stages. Stage 1 selects and fits the polynomial relationship between accuracy trust, favorability, and perceived capability. Those coefficients are then held fixed. Stage 2 uses work and demographic variables to predict the error left by Stage 1. Only the Stage 2 penalty and its contribution to the final prediction are retuned when the target metric changes from RMSE to MAE. Because Stage 1 does not include the context matrix or the Stage 2 tuning metric, its coefficients remain unchanged when either one changes. Both stages use ridge regression and source-year data.

We study the observed Stack Overflow survey rating of how well respondents believe their AI tools handle complex development tasks. This appraisal is available in both the 2024 and 2025 official surveys. Accuracy trust supplies the cognitive coordinate, and favorability toward AI tools supplies the affective coordinate. The estimand is perceived AI capability for complex tasks. This definition keeps the algorithmic claim aligned with the measured response.

Recent studies on human–AI collaboration distinguish evaluations of reliability and accuracy from emotional or affective responses to AI (Lee and See, 2004; Glikson and Woolley, 2020; Gillath et al., 2021; Riley and Dixon, 2024). A person may judge AI output as accurate while remaining unfavorable toward its use, or may express favorability despite limited confidence in its accuracy. Modeling both ratings and their polynomial terms preserves this distinction in a form that readers can inspect directly.

The article makes three contributions. First, TARS keeps the Stage 1 trust–favorability coefficients unchanged when the set of context variables or the Stage 2 tuning metric changes. Second, it is evaluated under a strict source-only protocol: all choices are made in 2024, predictions are stored before the 2025 outcome is accessed, and scoring follows an identifier-based join. Third, the evaluation compares TARS with a Stage 1-only model, one-stage models, and a model that reverses the two stages. It also tests four sets of context variables, separate RMSE and MAE tuning, computational cost, 20 source splits, large target groups, and 13 leave-one-group-out prediction tasks. Together, these tests ask whether one interpretable first-stage model can support different second-stage corrections and improve the metric that matters for a future survey wave, including groups absent from source fitting.

The measurement scope is defined at the item level. The accuracy item directly asks about trust in AI output accuracy; the affective item records favorability toward AI tools; and the outcome records perceived capability for complex tasks. Perceived usefulness, self-efficacy, creativity, adaptive performance, and realized job performance remain distinct constructs (Davis, 1989; Venkatesh et al., 2003; Compeau and Higgins, 1995). This alignment gives the predictive task a clear psychological meaning and a reproducible operational definition.

## 2 Theoretical framework

### 2.1 Cognitive trust and affective stance toward AI

Research on trust in automation emphasizes judgments of reliability, accuracy, transparency, competence, and task usefulness (Parasuraman and Riley, 1997; Jian et al., 2000). Reliance also varies with system behavior, experience, and operator expectations (Dzindolet et al., 2003; Lee and See, 2004; Hoff and Bashir, 2015; Glikson and Woolley, 2020). Affective processes, attachment-related cues, and emotional evaluations further shape responses to AI (Gillath et al., 2021; Riley and Dixon, 2024). Responses to algorithms are not uniformly favorable or unfavorable. Observed errors, advice framing, and perceived task subjectivity can shift reliance (Dietvorst et al., 2015; Logg et al., 2019; Castelo et al., 2019). This evidence motivates a two-coordinate representation: accuracy trust captures the cognitive appraisal of output quality, whereas favorability captures the affective stance toward using AI in the development workflow.

Accuracy trust should carry the stronger association because output accuracy and complex-task capability both concern tool competence. Favorability contributes a separate evaluative coordinate that may reflect willingness to engage with the tool, accumulated experience, or affective acceptance. TARS fits these terms first and reports their coefficients directly. Work and demographic variables enter only in the second-stage error correction.

### 2.2 Configuration and response-surface analysis

Congruence approaches represent two evaluations with polynomial terms rather than collapsing them into a single score (Edwards and Parry, 1993; Edwards and Cable, 2009). Cognitive–affective systems theory also emphasizes organized cognitive and affective units (Mischel and Shoda, 1995). These terms form Stage 1 of TARS. The line-of-congruence slope describes how perceived capability changes when both ratings rise together. The line-of-incongruence slope indicates which rating carries greater weight, and the curvature terms show whether the relationship departs from a plane. These parameters support substantive interpretation and allow the Stage 1 model to be checked directly.

Let *C*_*i*_ denote accuracy trust and *A*_*i*_ denote affective favorability, each centered at the scale midpoint of 3. We estimate the unconstrained polynomial model

$$\begin{array}{l} Y_{i}=b_{0}+b_{1}C_{i}+b_{2}A_{i}+b_{3}C_{i}^{2}+b_{4}C_{i}A_{i}+b_{5}A_{i}^{2}+\gamma^{⊤}{\text{x}}_{i}+\epsilon_{i}, \end{array}$$ (1)

where *Y*_*i*_ is perceived complex-task capability and **x**_*i*_ contains audited context controls. The line-of-congruence (LOC) slope and curvature are *a*_1_ = *b*_1_+*b*_2_ and *a*_2_ = *b*_3_+*b*_4_+*b*_5_. The line-of-incongruence (LOIC) slope and curvature are *a*_3_ = *b*_1_−*b*_2_ and *a*_4_ = *b*_3_−*b*_4_+*b*_5_ (Edwards and Parry, 1993). Evidence for a general mismatch penalty would require a negative LOIC curvature with an interval that excludes zero in both years. Predictive RSA features are evaluated separately from this inferential response-surface model.

The analysis asks five questions. First, does perceived capability rise when accuracy trust and favorability rise together? Second, is that joint increase curved rather than linear? Third, does disagreement between the two ratings produce a general mismatch penalty? Fourth, when the ratings differ, does accuracy trust or favorability have the stronger association? Fifth, does adding context in Stage 2 improve prediction beyond the trust–favorability terms? The fourth, directional comparison was not preregistered and is treated as exploratory.

## 3 Materials and methods

### 3.1 Data sources and design

The study uses the official respondent-level CSV files and schemas released for the Stack Overflow Developer Survey 2024 and 2025 (Stack Overflow, 2024, 2025). The 2024 file contains 65,437 retained responses and 114 columns; the 2025 file contains 49,191 retained responses and 172 columns. In both files, ResponseId is complete and unique. The official schemas show identical wording for AIComplex, AIAcc, AISent, and AIThreat across years. Stack Overflow recruited respondents through its own channels, making people who were highly engaged with the platform more likely to encounter the survey. These are not probability samples of developers or the general workforce.

This is a repeated cross-sectional secondary analysis of different respondents in the two survey years. Within-year evaluation uses a held-out subset of the 2024 responses, and cross-year evaluation trains on 2024 and evaluates on 2025.

### 3.2 Sample construction

For each year, respondents were retained in the following order: (a) selected “I am a developer by profession,” (b) met the year-specific employed-status criterion, (c) provided a valid response to the complex-task capability item, (d) provided a valid accuracy-trust response, and (e) provided an ordered favorability response. The employment criterion was “Employed, full-time” in 2024 and “Employed” in the redesigned 2025 item. The 2025 outcome response “I don't use AI tools for complex tasks / I don't know” falls outside the ordered outcome scale. The favorability response “Unsure” likewise falls outside its ordered scale. Both responses were treated as non-evaluable. No multivariate-outlier method, variable set, or threshold was applied. Accordingly, zero respondents were removed as multivariate outliers in 2024 and zero in 2025; all otherwise eligible observations were retained.

Table 1 reports the loss at every stage rather than only the retained totals. Requiring complete outcome, trust, and favorability items removed a large share of otherwise eligible respondents. The final samples contain 34.78% of the official 2024 records and 34.60% of the official 2025 records. These restrictions define the population to which the estimates apply. They may also create selection or collider bias if survey completion is jointly related to attitudes, tool use, occupation, or the outcome.

**Table 1:** Sample construction from the official survey files.

Filter stage	2024 (*n*)	2024 excluded, (*n*) (%)	2025 (*n*)	2025 excluded, (*n*) (%)	Inclusion/exclusion rationale
Official retained responses	65,437	–	49,191	–	Starting population in each official public file
Developer by profession	50,207	15,230 (23.27)	37,467	11,724 (23.83)	Define the professional-developer population
Employed professional developer	39,937	10,270 (20.46)	28,774	8,693 (23.20)	Apply the disclosed year-specific employment rule
Valid complex-task capability outcome	23,090	16,847 (42.18)	17,211	11,563 (40.19)	Require the observed outcome; exclude missing and the non-evaluable 2025 response
Valid cognitive-trust predictor	23,082	8 (0.03)	17,184	27 (0.16)	Require the ordered accuracy-trust item
Valid affective-stance predictor	22,757	325 (1.41)	17,021	163 (0.95)	Require an ordered favorability response; exclude missing and “Unsure”

Exclusion percentages use the immediately preceding row as the denominator.

### 3.3 Outcome definition and measurement properties

The outcome is the response to AIComplex, “How well do the AI tools you use in your development workflow handle complex tasks?” The five ordered responses were coded 1 (very poor), 2 (bad), 3 (neither good nor bad), 4 (good, but not great), and 5 (very well). Both official schemas use the same wording and evaluable category meanings. We analyze this observed ordinal item directly. Table 2 reports its distribution, central tendency, dispersion, and descriptive correlations.

**Table 2:** Observed properties of the final single-item outcome.

Property	2024	2025
Analytic *n*	22,757	17,021
Mean (SD)	2.798 (1.096)	2.666 (1.223)
Median (IQR)	3 (2)	3 (2)
Category 1, very poor	12.40%	21.87%
Category 2, bad	32.29%	27.47%
Category 3, neither	21.19%	16.97%
Category 4, good but not great	31.37%	29.63%
Category 5, very well	2.76%	4.07%
Spearman *r*_*s*_, accuracy trust	0.534	0.628
Spearman *r*_*s*_, affective stance	0.382	0.521
Spearman *r*_*s*_, perceived job threat	0.073	0.114

### 3.4 Complete variable and semantic mapping audit

Table 3 lists every raw field used in the analysis, its construct evidence, and the reason for its assigned role. The two main predictors and the outcome have identical wording and ordered response meanings across years. AI-use exposure is a close semantic match. The 2025 survey distinguishes daily, weekly, and less frequent current use; these categories were collapsed to the “current user” category represented by “Yes” in 2024. Because work-experience wording changed, this variable is used only as a context control with source-fitted median imputation and a missingness indicator. Explicit string rules mapped developer roles to six substantive families plus “other technical” and “missing.” Country values remained categorical, and the source-fitted encoder pooled categories occurring fewer than 100 times in the source fitting data.

**Table 3:** Full raw-variable and cross-year semantic audit.

Role	Raw field	Official wording / meaning	Coding and missing-data rule	Construct evidence and inclusion rationale	Cross-year decision
Outcome	AIComplex	Perceived handling of complex tasks	Five ordered categories coded 1–5; missing and non-evaluable 2025 category removed	Direct observed dependent variable; one anchor per year	Exact wording; common five-category scale
Cognitive predictor	AIAcc	Trust in accuracy of AI output	Highly distrust (1) to highly trust (5); missing removed	Only item that explicitly asks about trust; retained as accuracy trust	Exact wording and response meaning
Affective predictor	AISent	Favorability toward using AI tools	Very unfavorable (1) to very favorable (5); “Unsure” and missing removed	Direct favorability evidence defining affective stance	Exact wording and response meaning
Context	AISelect	Current AI-tool use and planned use	Current user / plans to use / does not plan / missing	AI-use exposure context	Close match; 2025 frequency collapsed
Context	AIThreat	Perceived threat to current job	No / not sure / yes / missing, nominal	Nominal threat context	Exact wording and response meaning
Context	WorkExp	Years of work experience	Numeric; source-fitted median imputation plus missingness indicator	Work-experience context	Partial wording match; control only
Context	Age	Respondent age group	Nominal; missing category retained	Demographic context	Same construct; category availability differs
Context	DevType	Current or principal job role	Audited rule-based role family; missing retained	Surveyed role context	Partial wording change; harmonized family
Context	Country	Country of residence	Nominal; source categories below 100 pooled	Geographic context and subgroup audit	Exact construct; source-fitted handling
Filter	MainBranch	Relationship to professional coding	Require “developer by profession”	Defines the professional-developer population	Exact substantive criterion
Filter	Employment	Current employment status	2024 full-time; 2025 employed	Defines the employed population	Partial redesign; year-specific criterion disclosed

AIComplex is the sole target, while AIAcc and AISent are the two main predictors. Their semantic proximity is reported as a construct-overlap consideration. AIThreat is a nominal context variable.

The role-family audit defined the following groups: software development (full-stack, back-end, front-end, desktop, mobile, game, embedded, and QA/test); data and AI (data engineer, data scientist, machine learning, AI/ML, developer-AI, and applied scientist); infrastructure and operations (DevOps, cloud infrastructure, system administration, site reliability, and support engineering); management and architecture (manager, executive, architect, project manager, product manager, and founder); research and education (academic, research, scientist, and educator); security; other technical; and missing. These rules were fixed before model fitting and applied identically to both years.

### 3.5 Preprocessing, feature sets, and models

Three nested feature sets were used. The context-only set contains work experience, AI-use exposure, perceived job threat, age group, role family, and country. The trust main-effects set adds centered accuracy trust (*C*) and affective stance (*A*). The RSA set adds *C*^2^, *CA*, and *A*^2^. Numeric variables were median-imputed from the source fitting data and standardized, with a missingness indicator added when needed. Categorical variables were one-hot encoded using only the source fitting data. A source-fitted infrequent-category rule handled unseen target categories.

All transformations were fitted within the information available to each protocol. The 2024 holdout inherited medians, scales, category levels, and pooling decisions from the 2024 training portion. The strict cross-year models inherited them from the complete 2024 source sample. The outcome, accuracy-trust item, and favorability item were not imputed. Item wording was retained exactly as published in the official survey files. The mappings in Table 3 use deterministic, inspectable string and category rules.

The comparison set includes a source-mean floor, context-only ridge, trust main-effects ridge, RSA ridge, elastic net (Zou and Hastie, 2005), random forest (Breiman, 2001), histogram gradient boosting (Friedman, 2001), and a multilayer perceptron (Rumelhart et al., 1986). The implementation used scikit-learn (Pedregosa et al., 2011). Pre-processing, tuning, and training use source-year records. Additional reproducibility specifications and analyses are provided in the Supplementary Appendix Tables 10–16.

### 3.6 Theory-anchored residual surface learning

Theory-guided data science can place domain knowledge inside a model rather than applying it only after fitting (Karpatne et al., 2017). TARS applies that principle to source-only learning for repeated surveys. Let ${\text{z}}_{i}=\left(C_{i},A_{i},C_{i}^{2},C_{i}A_{i},A_{i}^{2}\right)$ denote the Stage 1 trust–favorability terms, and let **x**_*i*_ denote the audited context features. TARS first estimates

$$\begin{array}{l} \hat{\text{θ}}=\arg\underset{\text{θ}}{\min}\underset{i}{\sum }{\left(Y_{i}-{\text{z}}_{i}^{⊤}\text{θ}\right)}^{2}+{\text{λ}}_{z}∥\text{θ}{∥}_{2}^{2}, \end{array}$$ (2)

then forms residuals $r_{i}=Y_{i}-{\text{z}}_{i}^{⊤}\hat{\text{θ}}$ and estimates

$$\begin{array}{l} \hat{\text{ϕ}}=\arg\underset{\text{ϕ}}{\min}\underset{i}{\sum }{\left(r_{i}-{\text{x}}_{i}^{⊤}\text{ϕ}\right)}^{2}+{\text{λ}}_{x}∥\text{ϕ}{∥}_{2}^{2}. \end{array}$$ (3)

The final score is ${\hat{Y}}_{i}={\text{z}}_{i}^{⊤}\hat{\text{θ}}+\gamma {\text{x}}_{i}^{⊤}\hat{\text{ϕ}}$. Freezing $\hat{\text{θ}}$ prevents the context variables in Stage 2 from changing the Stage 1 coefficients. Separate penalties control shrinkage for the five Stage 1 terms and the larger context set. The gate γ controls how much of the Stage 2 correction enters the final score. The source-year grid compared main-effect and polynomial Stage 1 models, λ_*z*_∈{0.1, 1, 10, 100}, λ_*x*_∈{1, 10, 100, 1000, 10000}, and γ∈{0, 0.25, 0.5, 0.75, 1}.

Figure 1 shows the two stages and the order of the source-only 2025 evaluation. Table 4 summarizes the training and source-only prediction procedure.

**Figure 1:**
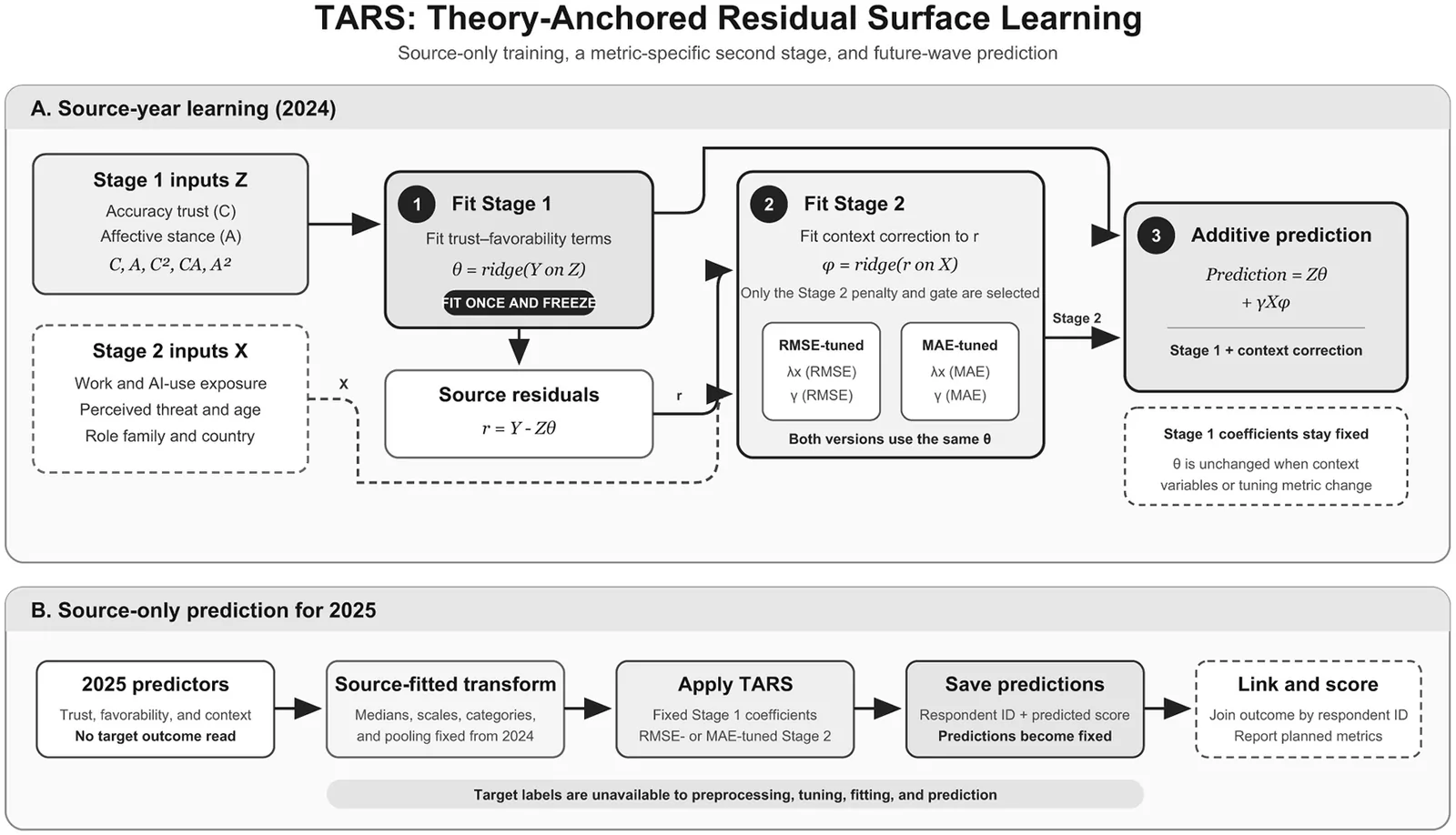
TARS architecture and source-only cross-year workflow. Stage 1 fits the relationship between accuracy trust, favorability, and perceived capability, and then holds those coefficients fixed. Stage 2 uses context variables to predict the remaining error and is tuned separately for RMSE or MAE. The 2025 predictors are transformed using rules learned from 2024, predictions are saved, and the 2025 outcome is then linked only for final evaluation.

**Table 4:** TARS training and source-only prediction procedure in plain language.

Step	Operation
1	Learn all data-cleaning and coding rules from 2024. Create one matrix for the trust–favorability terms (**Z**) and one for the context variables (**X**).
2	Use the 2024 validation set to choose Stage 1 and λ_*z*_. Fit Stage 1 and then keep $\hat{\text{θ}}$ fixed.
3	Calculate the prediction error left by Stage 1. For RMSE or MAE, choose only the Stage 2 penalty ${\text{λ}}_{x}^{\left(\ell \right)}$ and contribution γ^(ℓ)^.
4	Fit Stage 1 once using all 2024 responses, and fit the selected Stage 2 correction.
5	Apply the 2024 coding rules to the 2025 predictors and save all predictions before reading the 2025 outcome.
6	After predictions are saved, link the 2025 outcome by respondent identifier and calculate the planned metrics.

*Selecting Stage 2 for RMSE or MAE*. The Stage 1 model and λ_*z*_ are selected once using the primary squared-error criterion. With $\hat{\text{θ}}$ fixed, each tuning metric ℓ selects only the Stage 2 penalty and gate:

$$\begin{array}{l} \left({\text{λ}}_{x}^{\left(\ell \right)},\gamma^{\left(\ell \right)}\right)=\arg\underset{{\text{λ}}_{x},\gamma }{\min}\underset{i\in V}{\sum }L_{\ell }\left(Y_{i},{\text{z}}_{i}^{⊤}\hat{\text{θ}}+\gamma {\text{x}}_{i}^{⊤}{\hat{\text{ϕ}}}_{{\text{λ}}_{x}}\right), \end{array}$$ (4)

where V is the source validation set and ℓ∈{RMSE, MAE}. Stage 2 remains a ridge regression; the selected λ_*x*_ and γ depend on the stated metric. RMSE tuning selected λ_*x*_ = 100 and γ = 1. MAE tuning reused the same polynomial Stage 1 model and λ_*z*_ = 0.1, while selecting λ_*x*_ = 10 and γ = 1.

*Why the Stage 1 coefficients do not change*. For centered slope blocks with the intercept handled separately, the first-stage solution is $\hat{\text{θ}}={\left({\text{Z}}^{⊤}\text{Z}+{\text{λ}}_{z}\text{I}\right)}^{-1}{\text{Z}}^{⊤}\text{y}$. This equation contains neither the context matrix **X** nor the Stage 2 choices λ_*x*_, γ, and $L_{\ell }$. Changing the context design to X^*^, or changing the Stage 2 tuning metric, therefore updates only ${\hat{\text{ϕ}}}^{\left(\ell \right)}$, ${\text{λ}}_{x}^{\left(\ell \right)}$, and γ^(ℓ)^. The same $\hat{\text{θ}}$ is reused when the source rows, **Z**, and λ_*z*_ are fixed. In a joint model, by contrast, the Stage 1 coefficients can change with correlations between the two predictor sets and with whole-model retuning. For *K* tuning metrics, TARS fits Stage 1 once and selects *K* Stage 2 corrections. Under a dense primal ridge solve, each fitted version retains separate Stage 1 and context factorizations with $O\left(np_{z}^{2}+p_{z}^{3}+np_{x}^{2}+p_{x}^{3}\right)$ operations. Here *p*_*z*_ = 5, and the complete encoded context block has *p*_*x*_ = 66.

The procedure uses two standard ridge regressions fitted on the source year. Its distinguishing constraint is that the Stage 1 coefficients cannot be changed by the context variables or by the metric used to tune Stage 2. The information-access design matches prediction before a future survey outcome is available.

Four source-tuned comparisons test this constraint. The Stage 1-only model omitted the context correction. One-stage RSA estimated the trust–favorability and context terms together under one ridge penalty. A stronger joint model used separate penalties λ_*z*_ and λ_*x*_ but still estimated both predictor sets at the same time. It therefore matched TARS's shrinkage flexibility without fixing either set of coefficients. A reversed two-stage model fitted context first and then fitted the trust–favorability terms to the remaining error. The RMSE comparisons used the same 2024 split, validation criterion, feature mappings, 2025 predictor table, and bootstrap respondents as TARS-RMSE. The MAE comparison independently tuned TARS-MAE, one-stage RSA, and trust main effects by validation MAE on the same source split. These comparisons test whether fitting the trust–favorability terms first matters and whether Stage 2 can target a different error metric without changing them.

### 3.7 Inferential response-surface specification

The inferential polynomial model in Equation 1 was estimated separately in the complete 2024 (*n* = 22, 757) and 2025 (*n* = 17, 021) analytic samples. *C* and *A* were centered at their theoretical scale midpoint of 3. No observed-minimum or year-specific rescaling was used. Work experience was median-imputed within year and standardized. AI-use exposure, perceived job threat, age group, and role family were entered as indicator controls. Country was retained in the predictive models but omitted from the inferential RSA because hundreds of sparse country indicators would obscure the trust–favorability coefficients. Ordinary least squares was used with HC3 heteroskedasticity-consistent standard errors. The reference categories were “not sure” for perceived job threat, 18–24 years for age, and data/AI for role family. The 2025 AI-exposure reference was missing/uncodable. AI exposure had no variation in the 2024 outcome-complete sample and was therefore absent from that year's design matrix. Supplementary Appendix Table 12 reports all trust–favorability and control coefficients, standard errors, and confidence intervals.

### 3.8 Evaluation protocols and tuning

Cross-year survey prediction is difficult because the source and target waves may differ in predictor distributions and respondent composition (Pan and Yang, 2010; Ben-David et al., 2010). The temporal holdout and leave-one-group-out evaluations therefore use validation and test sets that reflect the intended use rather than assuming that all observations are interchangeable (Roberts et al., 2017).

The 2024 analytic sample was split, with outcome stratification, into 60% training, 20% validation, and 20% test sets using seed 20260823. Hyperparameters were selected by validation RMSE. For cross-year evaluation, the selected configuration was refit on all 2024 observations and evaluated on all eligible 2025 observations.

The strict source-only protocol fits every pre-processing operation and model using 2024 features and labels. The UDA protocol applies CORAL to align the covariance of source features with that of all unlabeled 2025 features (Sun and Saenko, 2016). CORAL is transductive because the full target feature distribution is available during fitting. Target labels remain reserved for final evaluation. Each algorithm is reported under both protocols, matching target-feature access within the UDA comparison.

The TARS comparison enforced source-only access in the order of file operations. Before AIComplex was read, a 2025 predictor table was built from branch, employment, trust, favorability, and context fields; this table contained 19,961 rows. Predictions from TARS and its two ridge references were then written to disk. The 2025 outcome was subsequently mapped and joined by the unique ResponseId, yielding 17,021 evaluable cases. This order provides a direct check that target outcomes were unavailable when predictions were made. The analysis code, data-download instructions, and reference outputs are provided in Supplementary Data Sheet 1.

One test changed only the set of context variables while holding the source rows, Stage 1 matrix, selected penalties, gate, target rows, and bootstrap respondents fixed. Four prespecified sets were fitted: work and AI-use context (15 encoded columns), demographic context (51), all non-geographic context (21), and the complete context set (66). For TARS and the joint model, the test records how much the Stage 1 coefficients change relative to the complete context set. It also records paired 2025 differences in *R*^2^, RMSE, MAE, and quadratic weighted kappa. This test directly checks whether the Stage 1 coefficients remain fixed when the context variables change.

The leave-one-group-out analysis tested prediction for a role or country group absent from source selection and fitting. The seven most frequent 2024 countries were defined from source features, with all remaining countries pooled. Role families and country buckets required at least 200 source observations and 200 target predictor rows. For each of the resulting 13 groups (five role families and eight country buckets), all members of that group were removed from the complete 2024 source sample. The remaining source rows were split 60/20/20 with the primary seed. TARS and one-stage RSA hyperparameters were reselected on these reduced training and validation sets, and the selected models were refitted on all remaining source rows. TARS-MAE and MAE-tuned RSA then predicted only the corresponding 2025 group. All predictions were stored before the 2025 outcome was read. This design prevents either model from using labeled 2024 members of the evaluated group during selection or fitting. It retains the same survey construct and cross-year outcome definition.

The strict source-only panel evaluates prospective prediction before target-year records are observed. The CORAL panel evaluates the transductive setting in which the unlabeled feature distribution of the same 2025 respondents is available after source fitting. Each panel is interpreted within its corresponding information-access regime.

The selected settings were as follows: context ridge α = 10; trust and RSA ridge α = 100; elastic net α = 0.001, *l*_1_ ratio 0.9; random forest with 250 trees, minimum leaf size 20, and 0.5 feature fraction; histogram gradient boosting with learning rate 0.03, 15 leaves, 300 iterations, and *l*_2_ = 1; and a one-hidden-layer multilayer perceptron with 32 units and α = 0.0001. TARS-RMSE selected the polynomial Stage 1 model, λ_*z*_ = 0.1, λ_*x*_ = 100, and γ = 1. TARS-MAE reused that Stage 1 model and λ_*z*_ while selecting λ_*x*_ = 10 and γ = 1. The MAE-tuned RSA and trust references selected α = 10 and α = 1, respectively. Stochastic models used seeds 101, 211, 307, 401, and 503. Their predictions were averaged across seeds.

Computational cost covered complete source pre-processing, model fitting, and prediction for all 19,961 rows in the 2025 predictor table. After one warm-up run, seven measured runs were recorded on the audited CPU environment. Every raw elapsed time was retained for verification. The benchmark used the selected production configuration, including five fitted estimators for the stochastic random-forest and multilayer-perceptron ensembles. Median time and the interquartile range are reported because elapsed time depends on the computing environment.

### 3.9 Uncertainty and statistical interpretation

Performance is summarized by *R*^2^, RMSE, and MAE under both within-year and cross-year protocols. Because the observed outcome is ordered, the TARS comparison also reports quadratic weighted kappa after rounding predictions to the nearest valid category. Ninety-five percent intervals were obtained from 1,000 respondent-level bootstrap resamples. The original model comparison used seed 260823; the independently locked TARS and model-comparison analyses used seeds 260831–260832; the RMSE-versus-MAE analyses used seeds 260833–260834; and the leave-one-group-out analysis used seed 260835 with task-specific offsets. Comparisons use the same resampled respondents for every model, preserving the pairing in reported difference intervals. A further source-only stability analysis repeated the 60/20/20 split for 20 fixed seeds. The performance tables do not report standard deviations across the five stochastic-model seeds. For stochastic models, predictions from seeds 101, 211, 307, 401, and 503 were averaged first; the reported point estimates and 95% intervals were then calculated from that averaged prediction. Thus, the displayed precision reflects respondent-level resampling, not five-seed variability. RSA coefficients were estimated separately by year using HC3 heteroskedasticity-consistent standard errors. Given the large samples, interpretation emphasizes effect size and interval width rather than isolated *p*-values. All interpretations remain observational.

For the target-group analysis, 2025 RMSE, MAE, and mean residual bias were calculated for TARS and RSA ridge in every role family with at least 200 observations. The analysis also included the seven largest country groups, with all remaining countries pooled. Positive bias denotes mean overprediction. TARS estimates, RSA estimates, and their paired differences use 1,000 within-group respondent bootstrap resamples with identical resampled respondents. This analysis tests whether the aggregate TARS error advantage extends across the major observed groups. The leave-one-group-out analysis applies the same paired resampling separately within each task after withholding its entire source group. It therefore tests prediction for groups absent from source fitting rather than subgroup performance after full-source fitting.

### 3.10 Use of generative AI during revision

Generative AI was not used to create or alter the survey data. During revision, ChatGPT was used for English-language polishing. OpenAI Codex (desktop application version 26.825.6671.0; GPT-5 model; OpenAI, San Francisco, CA, United States) assisted with code review, code organization, and assembly of the reproducibility package. For figure preparation, generative AI was used only to draft an initial version of the conceptual workflow figure; the final figure was independently created by the authors. All reported analyses were executed using the disclosed scripts and official public survey files, and the authors inspected the code and outputs.

## 4 Results

### 4.1 Sample and target audit

The complete audit retained 22,757 respondents in 2024 and 17,021 in 2025. Requiring a valid complex-task outcome produced the largest reduction. This step removed 42.18% of the preceding 2024 records and 40.19% of the preceding 2025 records. Fewer than 1.5% of outcome- and accuracy-complete cases were subsequently removed for the favorability item. The resulting population consists of employed professional developers who evaluated AI handling of complex tasks.

The five category percentages sum to 100% within rounding in each year. The mean outcome was 2.798 in 2024 and 2.666 in 2025, while both medians were 3. The lower 2025 mean coincided with a larger “very poor” category. We treat this cross-sample difference as descriptive because respondents and some response formats differ between years. Figure 2 shows the outcome-category distributions in the two analytic samples.

**Figure 2:**
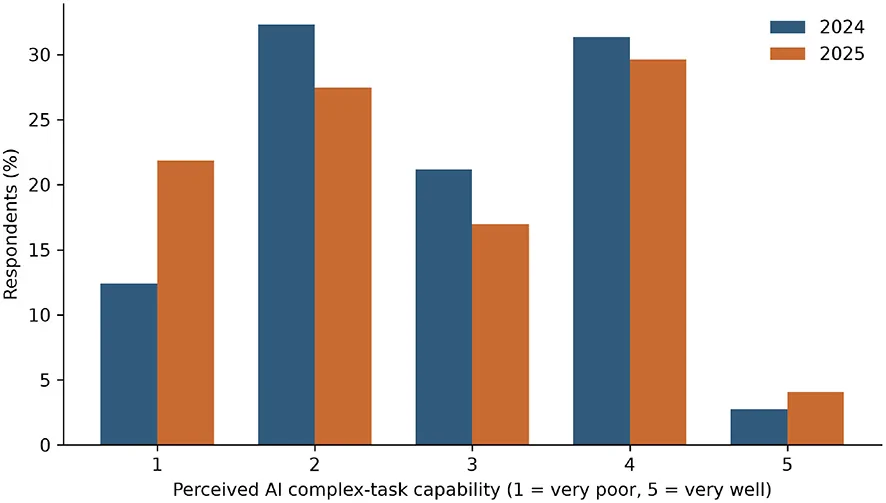
Distribution of the single-item perceived complex-task capability outcome. Percentages are computed within each analytic sample.

### 4.2 Source-year category-error stability

The 2024 holdout characterizes the source-year behavior of the proposed constraint (Table 5). TARS attained MAE 0.740, compared with 0.743 for one-stage RSA ridge, and increased quadratic weighted kappa from 0.465 to 0.477. Its *R*^2^ = 0.325 [0.301, 0.349] was within the leading model range. Across 20 additional source-only splits, TARS reduced MAE in 19 (mean difference −0.00147). This pattern identifies absolute category error as the stable source-year advantage of theory-anchored residual correction.

**Table 5:** Within-year 2024 holdout performance with 95% respondent-bootstrap intervals.

Model	*R* ^2^	RMSE	MAE
Source mean	0.000 [−0.001, 0.000]	1.096 [1.083, 1.110]	0.961 [0.947, 0.975]
Context-only ridge	0.048 [0.036, 0.061]	1.070 [1.055, 1.085]	0.929 [0.915, 0.943]
Trust main-effects ridge	0.325 [0.301, 0.349]	0.901 [0.882, 0.917]	0.744 [0.729, 0.758]
RSA ridge	0.326 [0.300, 0.349]	0.900 [0.882, 0.917]	0.743 [0.727, 0.757]
TARS	0.325 [0.301, 0.349]	0.901 [0.883, 0.918]	0.740 [0.724, 0.754]
Elastic net	0.325 [0.300, 0.349]	0.901 [0.883, 0.918]	0.743 [0.727, 0.757]
Random forest	0.321 [0.295, 0.346]	0.904 [0.886, 0.920]	0.743 [0.728, 0.757]
Histogram gradient boosting	0.327 [0.302, 0.350]	0.900 [0.882, 0.916]	0.741 [0.726, 0.755]
Multilayer perceptron	0.328 [0.303, 0.352]	0.899 [0.881, 0.915]	0.740 [0.725, 0.754]

### 4.3 Source-only cross-year prediction

Among the strict source-only models, TARS had the highest explained-variance point estimate and the lowest RMSE and MAE point estimates in the 2025 comparison (Table 6). It reached *R*^2^ = 0.445 [0.434, 0.456], RMSE 0.911 [0.902, 0.921], and MAE 0.742 [0.735, 0.751]. Against one-stage RSA ridge, the paired *R*^2^ gain was 0.00179 [0.00131, 0.00221], MAE improved by 0.00643, and quadratic weighted kappa increased from 0.573 to 0.584. Against trust main effects, the paired *R*^2^ gain was 0.00261 [0.00188, 0.00329]. These predictions were stored before the target outcome was accessed. The comparison supports the intended use: improving prediction for a future survey wave while keeping the Stage 1 trust–favorability coefficients fixed. Figure 3 compares the strict source-only and transductive CORAL results for the reference models. Figure 4 shows the paired *R*^2^ differences between TARS and the one-stage references.

**Table 6:** Protocol-matched 2024-to-2025 performance with 95% respondent-bootstrap intervals.

Model	*R* ^2^	RMSE	MAE
Strict source-only			
Context ridge	0.064 [0.058, 0.070]	1.184 [1.176, 1.192]	1.044 [1.035, 1.052]
Trust ridge	0.443 [0.431, 0.454]	0.913 [0.904, 0.923]	0.746 [0.738, 0.754]
RSA ridge	0.444 [0.432, 0.454]	0.913 [0.903, 0.922]	0.749 [0.741, 0.757]
TARS	0.445 [0.434, 0.456]	0.911 [0.902, 0.921]	0.742 [0.735, 0.751]
Elastic net	0.443 [0.432, 0.454]	0.913 [0.904, 0.922]	0.749 [0.741, 0.757]
Random forest	0.436 [0.425, 0.448]	0.918 [0.909, 0.928]	0.755 [0.747, 0.763]
Hist. gradient boosting	0.440 [0.429, 0.451]	0.915 [0.906, 0.925]	0.758 [0.749, 0.765]
Multilayer perceptron	0.442 [0.431, 0.453]	0.914 [0.904, 0.923]	0.744 [0.735, 0.752]
Transductive CORAL UDA			
Context ridge	0.063 [0.057, 0.070]	1.184 [1.176, 1.192]	1.044 [1.034, 1.052]
Trust ridge	0.407 [0.397, 0.418]	0.942 [0.933, 0.950]	0.796 [0.788, 0.803]
RSA ridge	0.403 [0.392, 0.413]	0.945 [0.936, 0.954]	0.796 [0.788, 0.804]
Elastic net	0.402 [0.392, 0.413]	0.946 [0.937, 0.955]	0.797 [0.788, 0.805]
Random forest	0.368 [0.360, 0.377]	0.972 [0.963, 0.980]	0.839 [0.831, 0.846]
Hist. gradient boosting	0.355 [0.346, 0.364]	0.982 [0.973, 0.990]	0.848 [0.840, 0.856]
Multilayer perceptron	0.418 [0.406, 0.429]	0.933 [0.924, 0.942]	0.775 [0.767, 0.783]

**Figure 3:**
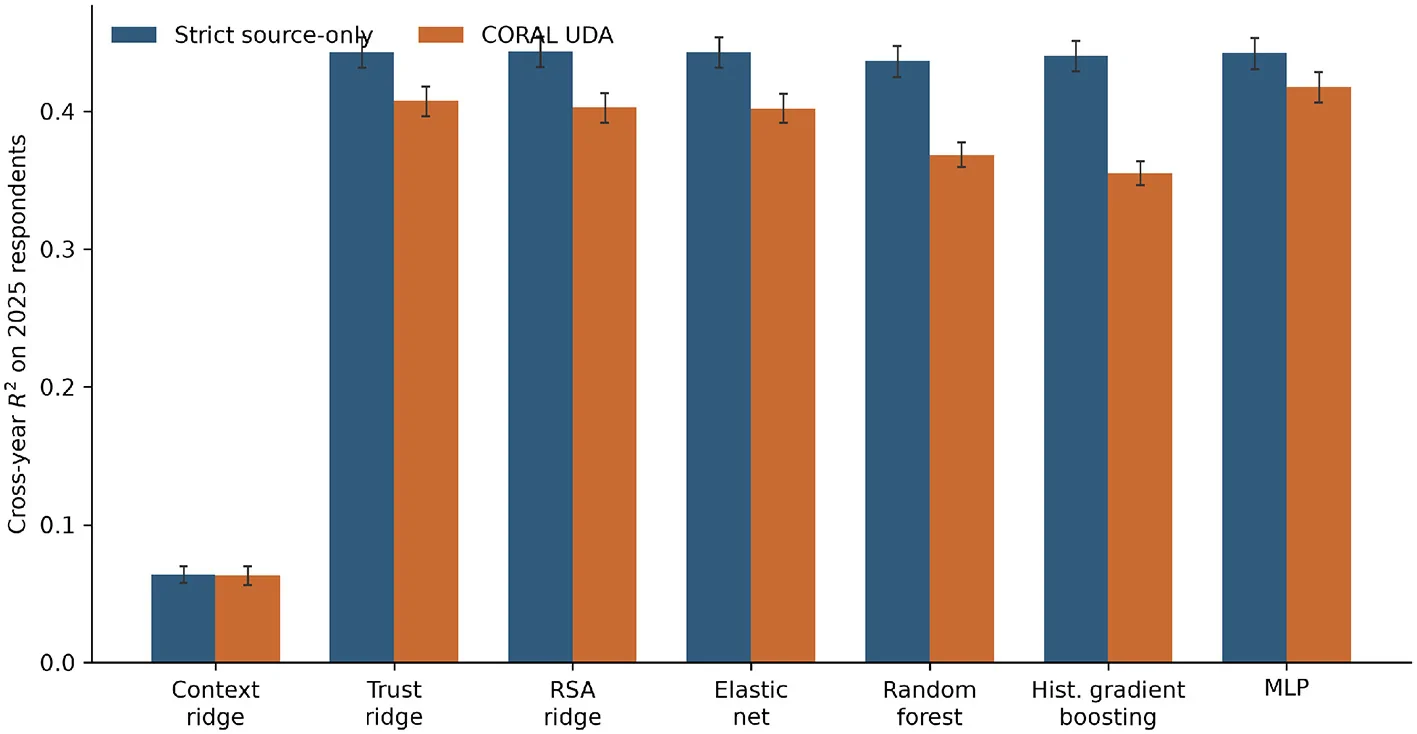
Strict source-only and transductive CORAL results on 2025 respondents. Error bars show 95% respondent-bootstrap intervals. Models are compared within the same feature-visibility protocol.

**Figure 4:**
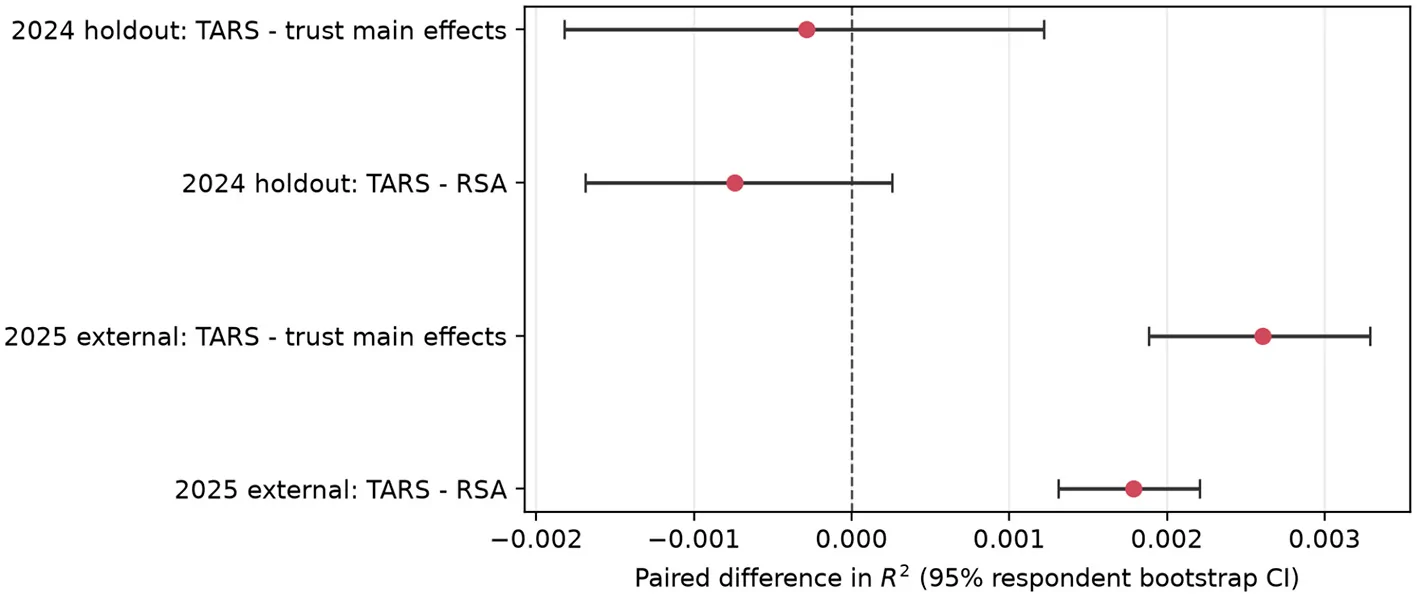
Paired TARS differences from the one-stage RSA and trust-main-effects references. Error bars are 95% respondent-bootstrap intervals; the vertical line marks no difference.

The transductive CORAL panel represents the condition in which the complete unlabeled target feature distribution is available during fitting. TARS addresses prospective source-only prediction. Separate panels attribute performance to the corresponding information-access regime.

The two evaluations address different parts of the claim. Repeated 2024 holdouts assess whether the MAE result depends on one split. The 2025 test assesses gains in *R*^2^, MAE, and ordinal agreement. Together, they show how the two-stage correction behaves both within the source year and in a future wave.

### 4.4 Retuning Stage 2 for RMSE or MAE

Supplementary Appendix Table 15 tests whether Stage 2 can be retuned without changing Stage 1. TARS-RMSE and TARS-MAE used exactly the same polynomial Stage 1 coefficients; the maximum absolute coefficient difference was 0. On the 2024 holdout, the MAE-tuned Stage 2 reduced MAE by 0.00156 [0.00068, 0.00238] relative to the RMSE-tuned version. On the 2025 test, it reduced MAE by 0.00134 [0.00102, 0.00168]. The paired *R*^2^ difference of −0.00005 [−0.00045, 0.00036] and RMSE difference of 0.00004 [−0.00030, 0.00037] show no resolved change in those two metrics.

Against MAE-tuned one-stage RSA on the 2025 target, TARS-MAE increased *R*^2^ by 0.00177 [0.00131, 0.00223], reduced RMSE by 0.00145 [0.00107, 0.00185], reduced MAE by 0.00649 [0.00610, 0.00689], and increased quadratic weighted kappa by 0.01015 [0.00800, 0.01233]. The corresponding comparison with MAE-tuned trust main effects favored TARS-MAE on all four metrics. Thus, the error metric used to select Stage 2 can change without changing the Stage 1 psychological coefficients.

### 4.5 Two-stage comparisons, response-surface evidence, and efficiency

Table 7 separates the effect of stage order from the effects of predictors and penalties. The closest comparison was a one-stage ridge model with separate penalties for the trust–favorability and context terms. It used the same selected polynomial terms, λ_*z*_ = 0.1, and λ_*x*_ = 100, but estimated both sets of coefficients at the same time. TARS improved *R*^2^ over this model by 0.00156 [0.00113, 0.00194], reduced MAE by 0.00577 [0.00539, 0.00612], and increased quadratic weighted kappa by 0.01028 [0.00825, 0.01254]. Reversing the order, so that context was fitted first, produced paired TARS gains of 0.00483 [0.00335, 0.00630] in *R*^2^, 0.00617 [0.00506, 0.00735] in MAE reduction, and 0.01935 [0.01561, 0.02311] in kappa. The Stage 1-only comparison showed that adding Stage 2 supplied 0.01012 [0.00812, 0.01217] in *R*^2^ and reduced MAE by 0.01462 [0.01286, 0.01630]. Across all four comparisons, TARS produced the highest point *R*^2^ and kappa and the lowest RMSE and MAE. These results support fitting and fixing the trust–favorability terms before adding context.

**Table 7:** Two-stage and one-stage comparisons on 2025.

Model	*R*^2^ [95% CI]	RMSE [95% CI]	MAE [95% CI]	Quadratic weighted kappa [95% CI]
Stage 1 only	0.435 [0.425, 0.446]	0.919 [0.911, 0.929]	0.757 [0.750, 0.765]	0.582 [0.573, 0.591]
One-stage RSA ridge	0.444 [0.433, 0.454]	0.913 [0.904, 0.922]	0.749 [0.741, 0.757]	0.573 [0.564, 0.582]
One-stage ridge, separate penalties	0.444 [0.433, 0.455]	0.912 [0.904, 0.922]	0.748 [0.740, 0.756]	0.573 [0.564, 0.582]
Context-first two-stage model	0.440 [0.429, 0.451]	0.915 [0.906, 0.924]	0.749 [0.741, 0.756]	0.564 [0.555, 0.573]
TARS	0.445 [0.434, 0.456]	0.911 [0.902, 0.921]	0.742 [0.735, 0.751]	0.584 [0.574, 0.592]

Entries are point estimates and 95% respondent-bootstrap intervals. All models were selected using only 2024 validation RMSE.

Supplementary Appendix Table 14 reports the test that changes the context variables. Across the work and AI-use, demographic, non-geographic, and complete context sets, the five TARS Stage 1 coefficients remained exactly identical. Relative to the complete set, the one-stage separate-penalty model changed a Stage 1 coefficient by as much as 0.0202. Its maximum relative ℓ_2_ change was 4.04%, and the maximum change in the Stage 1 contribution to target predictions was 0.0488. TARS also improved *R*^2^, RMSE, MAE, and quadratic weighted kappa over that one-stage model for every context set; all 16 paired 95% intervals favored TARS. The gains ranged from 0.00036 to 0.00156 in *R*^2^, 0.00030 to 0.00128 in RMSE reduction, 0.00113 to 0.00577 in MAE reduction, and 0.00250 to 0.01372 in kappa. The fixed Stage 1 coefficients therefore coincided with predictive gains across the tested context sets.

The two-stage design retained the computational cost of linear regression. In seven measured runs after one warm-up, complete source pre-processing, fitting, and prediction of 19,961 target rows required a median 0.312 s (IQR 0.018), compared with 0.294 s for one-stage RSA. In the audited environment, TARS was 3.6 times faster than histogram gradient boosting, 28.5 times faster than the five-model multilayer-perceptron ensemble, and 41.7 times faster than the five-model random-forest ensemble. The tests therefore use two transparent ridge stages with subsecond runtime.

The trust–favorability model showed the same directional ordering across years (Table 8; Figure 5). The line-of-congruence slope was positive in 2024 (0.702, 95% CI [0.684, 0.720]) and 2025 (0.792 [0.775, 0.809]). Perceived capability therefore rose as accuracy trust and favorability increased together. The positive line-of-incongruence slope in both years indicates that accuracy trust carried the larger contribution when the ratings differed. The year-specific models explained 32.7 and 44.6% of observed outcome variance. The curvature estimates describe the remaining shape. These positive slopes are the main psychological relationship retained in Stage 1.

**Table 8:** Trust–favorability response-surface coefficients and derived parameters with HC3 standard errors and 95% confidence intervals.

Parameter	2024	2025
Panel A. Polynomial coefficients, estimate (SE) [95% CI]		
Intercept, *b*_0_	2.617 (0.034) [2.551, 2.683]	2.249 (0.271) [1.718, 2.780]
Cognitive trust, *b*_1_	0.476 (0.013) [0.451, 0.500]	0.501 (0.014) [0.473, 0.529]
Affective stance, *b*_2_	0.226 (0.015) [0.198, 0.255]	0.291 (0.014) [0.263, 0.319]
*C*^2^, *b*_3_	−0.002 (0.007) [−0.015, 0.011]	0.024 (0.008) [0.009, 0.039]
*C*×*A*, *b*_4_	0.019 (0.010) [−0.000, 0.039]	0.033 (0.011) [0.012, 0.055]
*A*^2^, *b*_5_	0.014 (0.008) [−0.001, 0.029]	0.021 (0.007) [0.006, 0.035]
Model *R*^2^ (adjusted *R*^2^)	0.327 (0.327)	0.446 (0.445)
Panel B. Derived surface parameters, estimate [95% CI]		
LOC slope, *a*_1_	0.702 [0.684, 0.720]	0.792 [0.775, 0.809]
LOC curvature, *a*_2_	0.0307 [0.0182, 0.0433]	0.0780 [0.0655, 0.0905]
LOIC slope, *a*_3_	0.249 [0.199, 0.299]	0.210 [0.157, 0.263]
LOIC curvature, *a*_4_	−0.0079 [−0.0474, 0.0316]	0.0115 [−0.0324, 0.0555]

**Figure 5:**
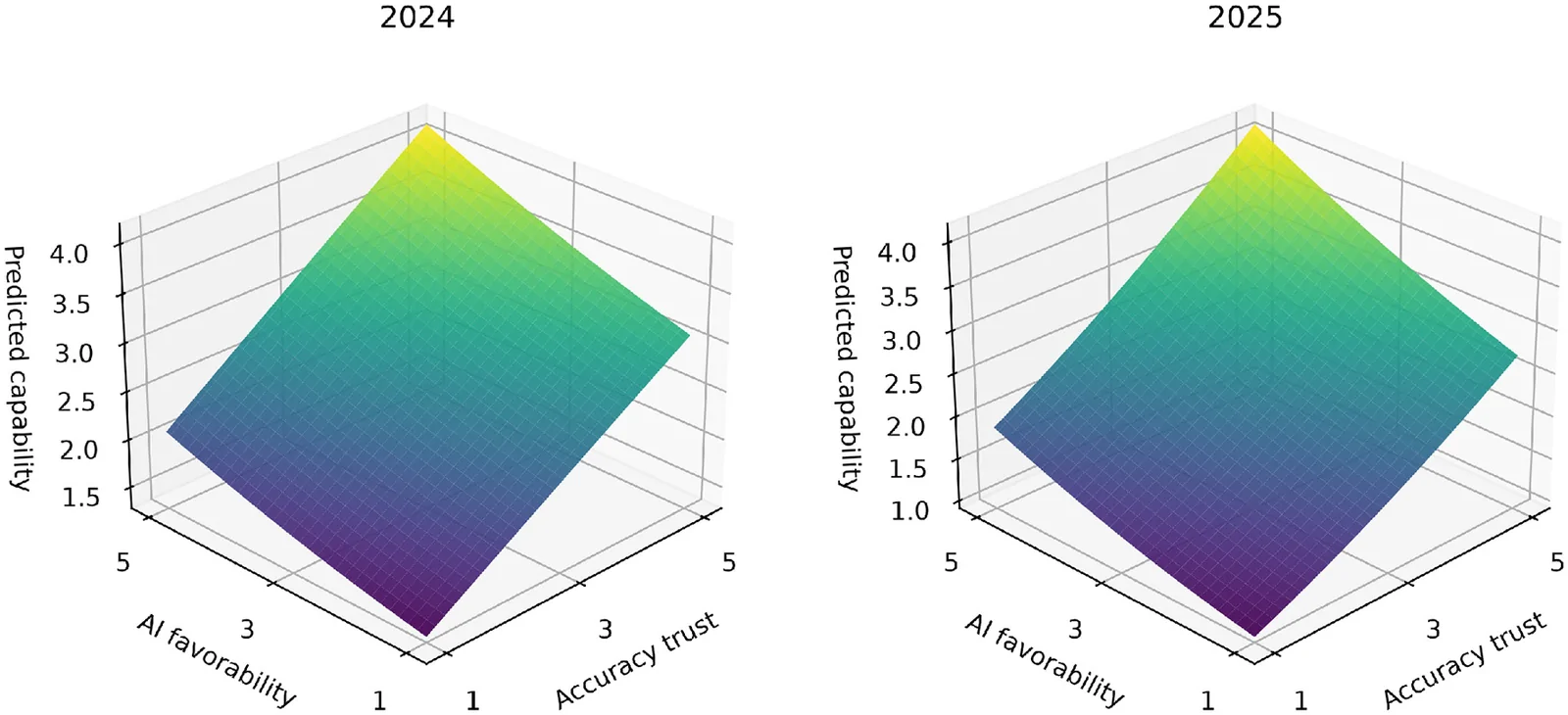
Year-specific polynomial response surfaces with context controls fixed at their reference values. Surface shape is descriptive.

### 4.6 Target-group error stability

The external-test differences were not confined to the aggregate sample. TARS achieved lower RMSE and MAE point estimates than one-stage RSA in all 13 audited role and country groups (Table 9). The paired 95% MAE-difference interval was below zero in every group, whereas the paired RMSE-difference interval was below zero in seven groups. MAE reductions ranged from 0.00421 to 0.01106, with the largest reduction in the other-technical role group. The pattern included the broad software-development group and every reported country grouping. Supplementary Appendix Table 13 reports signed calibration estimates and intervals separately.

**Table 9:** Target-group error stability in 2025.

Type	Group	*n*	TARS RMSE [95% CI]	RSA RMSE [95% CI]	Paired ΔRMSE [95% CI]	TARS MAE [95% CI]	RSA MAE [95% CI]	Paired ΔMAE [95% CI]
Role	Data and AI	1,060	0.935 [0.900, 0.971]	0.938 [0.904, 0.973]	−0.00269 [−0.00417, −0.00117]	0.771 [0.740, 0.802]	0.778 [0.747, 0.809]	−0.00700 [−0.00844, −0.00536]
Role	Infrastructure and operations	654	0.917 [0.868, 0.962]	0.919 [0.870, 0.963]	−0.00118 [−0.00313, 0.00075]	0.741 [0.698, 0.780]	0.747 [0.705, 0.786]	−0.00616 [−0.00813, −0.00421]
Role	Management and architecture	2,036	0.916 [0.890, 0.944]	0.919 [0.892, 0.946]	−0.00219 [−0.00324, −0.00123]	0.750 [0.726, 0.775]	0.757 [0.733, 0.781]	−0.00675 [−0.00787, −0.00565]
Role	Other technical	385	0.893 [0.826, 0.955]	0.898 [0.831, 0.959]	−0.00461 [−0.00736, −0.00211]	0.721 [0.667, 0.775]	0.732 [0.679, 0.785]	−0.01106 [−0.01373, −0.00831]
Role	Software development	12,653	0.908 [0.898, 0.920]	0.909 [0.899, 0.921]	−0.00121 [−0.00165, −0.00075]	0.740 [0.730, 0.750]	0.746 [0.737, 0.756]	−0.00623 [−0.00666, −0.00578]
Country	United States of America	3,528	0.915 [0.895, 0.935]	0.916 [0.896, 0.936]	−0.00105 [−0.00182, −0.00017]	0.739 [0.721, 0.756]	0.747 [0.729, 0.764]	−0.00771 [−0.00860, −0.00682]
Country	Germany	1,512	0.859 [0.830, 0.888]	0.860 [0.832, 0.889]	−0.00081 [−0.00209, 0.00042]	0.701 [0.675, 0.725]	0.707 [0.681, 0.731]	−0.00594 [−0.00721, −0.00464]
Country	India	1,226	0.894 [0.860, 0.929]	0.897 [0.863, 0.931]	−0.00285 [−0.00415, −0.00162]	0.725 [0.695, 0.753]	0.731 [0.701, 0.759]	−0.00610 [−0.00729, −0.00498]
Country	United Kingdom	921	0.903 [0.864, 0.940]	0.905 [0.866, 0.941]	−0.00116 [−0.00282, 0.00037]	0.736 [0.701, 0.768]	0.743 [0.710, 0.775]	−0.00723 [−0.00910, −0.00565]
Country	France	609	0.904 [0.857, 0.950]	0.906 [0.860, 0.951]	−0.00194 [−0.00380, 0.00018]	0.746 [0.707, 0.789]	0.752 [0.714, 0.795]	−0.00664 [−0.00861, −0.00447]
Country	Canada	602	0.918 [0.870, 0.967]	0.918 [0.872, 0.966]	−0.00039 [−0.00263, 0.00184]	0.745 [0.702, 0.790]	0.751 [0.709, 0.796]	−0.00631 [−0.00834, −0.00411]
Country	Ukraine	536	0.947 [0.896, 0.998]	0.948 [0.898, 0.998]	−0.00133 [−0.00320, 0.00049]	0.793 [0.748, 0.838]	0.797 [0.751, 0.842]	−0.00421 [−0.00620, −0.00232]
Country	Other countries	8,087	0.920 [0.906, 0.934]	0.922 [0.908, 0.935]	−0.00165 [−0.00218, −0.00111]	0.751 [0.740, 0.763]	0.757 [0.746, 0.769]	−0.00605 [−0.00657, −0.00551]

Entries are point estimates and 95% within-group respondent-bootstrap intervals. Paired differences are TARS minus RSA; negative values favor TARS.

The leave-one-group-out analysis repeated the comparison after removing each role or country group before source tuning and fitting (Supplementary Appendix Table 16). Across all 13 tasks, TARS-MAE produced lower MAE than the independently MAE-tuned one-stage RSA model, and every paired MAE interval was below zero. The median MAE reduction was 0.00793, with taskwise reductions from 0.00270 to 0.01635. TARS-MAE also produced lower RMSE in 11 tasks; 10 paired RMSE intervals were below zero. The analysis includes complete exclusion of the broad software-development role family and complete exclusion of all countries outside the seven source-defined major-country buckets. The MAE advantage therefore remains when the evaluated group contributes no labeled member to source selection or fitting.

## 5 Discussion

### 5.1 Principal findings

This study examines a specific role for theory-guided learning in repeated cross-sectional surveys. TARS fits the trust–favorability relationship first, keeps its coefficients fixed, and then uses context to correct the remaining prediction error. It applies this two-stage model to a future wave under strict source-only access. When the source data and Stage 1 design are fixed, changing the context variables or the Stage 2 tuning metric does not change the Stage 1 coefficients. The method therefore connects an interpretable psychological relationship with three practical requirements: preventing context from redefining that relationship, tuning prediction for a stated metric, and making predictions before target-year outcomes exist.

In the 2025 comparison, TARS had the highest *R*^2^ and quadratic weighted kappa point estimates and the lowest RMSE and MAE point estimates among the source-only models. The joint and reversed-order comparisons show the value of fitting and fixing Stage 1 before context correction. Tests using four context sets confirm that the Stage 1 coefficients stay fixed. Retuning Stage 2 for MAE improved absolute error without changing any Stage 1 coefficient or producing a resolved change in squared-error performance. The MAE advantage also appeared across 19 of 20 additional source holdouts, every one of the 13 audited large target groups, and all 13 independently tuned leave-one-group-out tasks. The two-stage fit remained subsecond and substantially faster than the non-linear reference models. These results address model structure, prediction error, runtime, split sensitivity, and group exclusion under the same source-only design.

TARS uses two ridge regressions. Stage 1 estimates the theoretically meaningful trust–favorability relationship. Stage 2 models contextual variation, with its penalty and contribution selected separately for RMSE or MAE. The joint and context-first comparisons test whether fitting Stage 1 first explains the difference. Because the Stage 1 equation does not contain the context matrix or the Stage 2 tuning metric, either can change while the Stage 1 coefficients remain fixed. The two ridge stages separate the relationship reported for interpretation from the correction used for prediction.

The response-surface terms supply the substantive interpretation. Jointly higher accuracy trust and favorability corresponded to higher perceived capability in both years, and accuracy trust contributed more strongly when the ratings differed. TARS keeps this ordering in Stage 1 while allowing work and demographic context to improve prediction in Stage 2. The source-only error results provide the principal evidence, with protocol and calibration analyses as complementary checks.

### 5.2 Theoretical implications

The study gives cognitive–affective organization a direct role in model construction. TARS does not estimate the trust and favorability terms only for interpretation after fitting. It estimates them first and keeps them fixed. Accuracy trust and favorability retain separate psychological meanings while contributing to one transparent part of the prediction. This design applies when a small, theory-defined predictor set must remain interpretable alongside a larger context set.

The cross-year estimates refine the substantive result. Perceived AI capability rose when accuracy trust and favorability increased together, and the stronger accuracy-trust contribution appeared in both waves. The approximately flat line-of-incongruence curvature indicates that the main information lies in the level and directional slopes. This result supports a simple Stage 1 model aligned with the observed tool appraisals.

### 5.3 Practical implications

TARS is suited to annual surveys, monitoring systems, and repeated organizational assessments in which future outcomes are not yet available, the trust–favorability relationship must remain interpretable, and computation is limited. Standard linear-model software can implement both ridge stages, and the order of prediction and outcome access can be checked directly. With the source data and Stage 1 model fixed, context fields can be added or removed without changing the Stage 1 coefficients. An organization can tune Stage 2 for RMSE when tracking a continuous score or for MAE when absolute category error is more important. Either choice leaves the psychological coefficients reported to stakeholders unchanged. In the seven-run complete fit-and-predict benchmark, TARS-RMSE remained subsecond. It also had the highest explained-variance point estimate and the lowest RMSE and MAE point estimates in the source-only 2025 comparison while using a small fraction of the time required by the evaluated tree and neural ensembles. For AI-governance use, accuracy judgments and affective stance should be recorded separately and interpreted alongside task-specific evidence about actual system performance. The resulting scores characterize appraisals of AI tools and are appropriate for aggregate monitoring, not employee selection or performance management.

### 5.4 Limitations

The data and task definition determine the scope conditions. The surveys are self-selected and developer-centered. The analytic population consists of employed professional developers who completed the three required items. The outcome, accuracy trust, and favorability measures are single self-reports collected in the same response. Their temporal order is therefore unknown, and the results concern association with and prediction of perceived AI capability rather than a longitudinal effect. Approximately 35% of each official file met the professional-status, employment, and complete-item criteria. Restricting the sample to complete outcome, trust, and favorability responses can create selection or collider bias if both AI attitudes or tool use and perceived capability affect whether those items are answered. Conditioning on professional status and employment can likewise induce associations among occupation, AI use, and the outcome within the selected sample. The estimates therefore describe eligible complete responders, not all survey respondents or workers. The current analysis does not use O^*^NET matching and does not delete multivariate outliers, so neither is a current selection criterion.

The outcome is modeled as an ordered five-category score. TARS therefore reports continuous-error metrics and quadratic weighted kappa, with predictions rounded only for the ordinal agreement calculation. The 2024 and 2025 samples contain different respondents, making the design repeated cross-sectional rather than individual-longitudinal. Although the outcome item wording and the five evaluable categories are the same, the 2025 survey adds a non-evaluable outcome response and redesigns the employment item. Observed cross-year distribution differences may therefore reflect instrument format and selection changes as well as differences in respondents' appraisals; they are not interpreted as psychological change over time. CORAL represents the transductive comparison condition, whereas a separate source-only protocol supports the TARS claim. The target-group and leave-one-group-out analyses cover large observed role and country groups within the same public survey program. They test prediction for groups under a common item definition, not transfer to a different population or measurement instrument. These conditions define the population, access regime, and inferential level to which the results apply.

Future work can apply the same two-stage structure to validated multi-item constructs, preregistered response-surface hypotheses, behavioral outcomes, other source-only adaptation methods, and repeated measures. Such designs would test whether fixing a theory-defined first stage is useful beyond perceived AI capability in developer surveys.

## 6 Conclusion

TARS addresses a focused cross-year prediction problem: keeping an interpretable trust–favorability relationship, using context to improve prediction, tuning for a stated error metric, and generating future-wave predictions before outcomes are available. It fits and fixes the trust–favorability terms in Stage 1, then tunes the context correction in Stage 2. The four-context-set test returned exactly the same Stage 1 coefficients. Retuning Stage 2 from RMSE to MAE also left those coefficients unchanged; the MAE-tuned version reduced target absolute error without a resolved change in squared-error performance. In the strict source-only 2025 comparison, TARS had the highest explained-variance point estimate and the lowest RMSE and MAE point estimates. It also reduced MAE in 19 of 20 additional source holdouts and lowered RMSE and MAE in every audited large target group. After each of 13 role or country groups was removed before source selection and fitting, TARS-MAE lowered absolute error in every corresponding 2025 task, with all 13 intervals favoring TARS. It also lowered RMSE in 11 tasks, with 10 intervals favoring TARS. The two ridge stages retained subsecond cost while running substantially faster than the evaluated non-linear baselines. For repeated AI-appraisal surveys, the method provides a clear first-stage psychological model, a metric-specific second-stage correction, a source-only access protocol, and prediction results supported by model comparisons, runtime tests, repeated splits, and leave-one-group-out evaluations.

## Data Availability

The datasets analyzed in this study are publicly available from the official Stack Overflow Developer Survey releases for 2024 and 2025 (Stack Overflow, 2024, 2025). Analysis code, data-download instructions, and reference outputs are provided in Supplementary Data Sheet 1. Additional reproducibility specifications and analyses are provided in the Supplementary Appendix. Further inquiries can be directed to the corresponding author.
