# Supplementary material for: Source-only cross-year prediction of perceived AI capability with a theory-anchored, loss-specific two-stage model
Source: Front Psychol. 2026 Oct 21;17:1867910. doi: 10.3389/fpsyg.2026.1867910 (PMC13730845; doi:10.3389/fpsyg.2026.1867910)
Supplement: Supplementary file 1 [file Supplementary_file_1.pdf]

# Supplementary Appendix

## Reproducibility Specifications

**Table 10.** Hyperparameter search space and selected configuration.

| Model | Source-year search space | Selected configuration |
|---|---|---|
| Ridge models | $\alpha \in \{0.1,1,10,100\}$ | Context: 10; trust and RSA: 100 |
| TARS-RMSE / TARS-MAE | Shared Stage 1: main or polynomial; $\lambda_z \in \{0.1,1,10,100\}$; Stage 2-specific $\lambda_x \in \{1,10,100,1000,10000\}$, $\gamma \in \{0,0.25,0.5,0.75,1\}$ | Shared polynomial Stage 1, $\lambda_z = 0.1$; RMSE: $\lambda_x = 100$, $\gamma = 1$; MAE: $\lambda_x = 10$, $\gamma = 1$ |
| Stage 1 only | Stage 1: main or polynomial; $\lambda_z \in \{0.1,1,10,100\}$ | Polynomial; $\lambda_z = 0.1$ |
| One-stage ridge, separate penalties | Stage 1: main or polynomial; $\lambda_z \in \{0.1,1,10,100\}$; $\lambda_x \in \{1,10,100,1000,10000\}$ | Polynomial; $\lambda_z = 0.1$; $\lambda_x = 100$ |
| Context-first two-stage model | Stage 1: main or polynomial; $\lambda_z \in \{0.1,1,10,100\}$; $\lambda_x \in \{1,10,100,1000,10000\}$; $\gamma \in \{0,0.25,0.5,0.75,1\}$ | Polynomial; $\lambda_z = 0.1$; $\lambda_x = 1000$; $\gamma = 1$ |
| Elastic net | $\alpha \in \{0.001,0.01,0.1\}$; $l_1$ ratio $\in \{0.1,0.5,0.9\}$ | $\alpha = 0.001$, ratio 0.9 |
| Random forest | 250 trees; minimum leaf $\in \{5,20\}$; feature fraction $\in \{0.5,1.0\}$ | Leaf 20; fraction 0.5 |
| Histogram gradient boosting | learning rate $\in \{0.03,0.06\}$; leaves $\in \{15,31\}$; $l_2 \in \{0,1\}$; 300 iterations | 0.03; 15 leaves; $l_2 = 1$ |
| Multilayer perceptron | hidden layers $\in \{(32),(64,32)\}$; $\alpha \in \{0.0001,0.001\}$ | (32); $\alpha = 0.0001$ |

The primary split seed was 20260823. Stochastic model seeds were 101, 211, 307, 401, and 503. The original comparison used bootstrap seed 260823. The TARS holdout and external model comparisons used seeds 260831 and 260832; the RMSE-versus-MAE source and target comparisons used seeds 260833 and 260834; and the target-group comparison used seed 260833. Each bootstrap used 1,000 respondent-level resamples. Validation RMSE selected the shared Stage 1 model and the RMSE-tuned Stage 2 correction. Validation MAE selected only the MAE-tuned Stage 2 correction and its MAE-tuned references. The TARS split-sensitivity analysis used 20 additional fixed split seeds (20260801-20260820). For every final cross-year comparison, 2025 predictions were saved before the outcome field was read or used to determine the evaluated subset.

**Table 11.** Computational efficiency for complete source preprocessing, fitting, and prediction of 19,961 rows in the 2025 predictor table. Times are medians and interquartile ranges from seven measured runs after one warm-up run on the audited CPU environment.

| Model | Fitted estimators | Median seconds | IQR seconds | Time relative to TARS |
|---|---|---|---|---|
| TARS | 2 | 0.312 | 0.018 | 1.0 |
| One-stage RSA ridge | 1 | 0.294 | 0.007 | 0.9 |
| Elastic net | 1 | 0.478 | 0.016 | 1.5 |
| Histogram gradient boosting | 1 | 1.115 | 0.087 | 3.6 |
| Multilayer perceptron | 5 | 8.899 | 0.120 | 28.5 |
| Random forest | 5 | 12.996 | 0.469 | 41.7 |

**Table 12.** Complete year-specific inferential RSA coefficients. Entries are estimate (HC3 SE) and 95% CI.

| **Term** | **2024 estimate (SE)** | **2024 95% CI** | **2025 estimate (SE)** | **2025 95% CI** |
|---|---|---|---|---|
| Intercept | 2.617 (0.034) | [2.551, 2.683] | 2.249 (0.271) | [1.718, 2.780] |
| Cognitive trust, C | 0.476 (0.013) | [0.451, 0.500] | 0.501 (0.014) | [0.473, 0.529] |
| Affective stance, A | 0.226 (0.015) | [0.198, 0.255] | 0.291 (0.014) | [0.263, 0.319] |
| $C^2$ | -0.002 (0.007) | [-0.015, 0.011] | 0.024 (0.008) | [0.009, 0.039] |
| $C \times A$ | 0.019 (0.010) | [-0.000, 0.039] | 0.033 (0.011) | [0.012, 0.055] |
| $A^2$ | 0.014 (0.008) | [-0.001, 0.029] | 0.021 (0.007) | [0.006, 0.035] |
| Work experience (standardized) | 0.004 (0.008) | [-0.012, 0.019] | -0.032 (0.016) | [-0.063, -0.002] |
| Job threat: missing | 0.073 (0.048) | [-0.022, 0.168] | 0.107 (0.434) | [-0.743, 0.957] |
| Job threat: no | -0.063 (0.016) | [-0.094, -0.032] | -0.067 (0.018) | [-0.102, -0.033] |
| Job threat: yes | 0.049 (0.023) | [0.004, 0.095] | 0.074 (0.024) | [0.026, 0.121] |
| Age: 25-34 | -0.027 (0.018) | [-0.062, 0.008] | -0.007 (0.026) | [-0.057, 0.043] |
| Age: 35-44 | -0.032 (0.021) | [-0.074, 0.009] | 0.018 (0.031) | [-0.044, 0.080] |
| Age: 45-54 | -0.002 (0.030) | [-0.061, 0.057] | 0.103 (0.045) | [0.014, 0.192] |
| Age: 55-64 | 0.119 (0.045) | [0.031, 0.207] | 0.254 (0.062) | [0.132, 0.377] |
| Age: 65 or older | 0.036 (0.105) | [-0.169, 0.241] | 0.462 (0.106) | [0.254, 0.670] |
| Age: prefer not to say | -0.079 (0.189) | [-0.450, 0.291] | 0.378 (0.188) | [0.009, 0.746] |
| Age: under 18 | 0.308 (0.168) | [-0.021, 0.637] | - | - |
| Role: infrastructure/operations | -0.038 (0.041) | [-0.119, 0.042] | -0.108 (0.046) | [-0.198, -0.018] |
| Role: management/architecture | -0.081 (0.037) | [-0.154, -0.009] | -0.009 (0.036) | [-0.079, 0.061] |
| Role: missing | 0.022 (0.044) | [-0.063, 0.108] | - | - |
| Role: other technical | -0.015 (0.041) | [-0.096, 0.065] | 0.009 (0.053) | [-0.096, 0.114] |
| Role: research/education | -0.051 (0.048) | [-0.144, 0.043] | 0.077 (0.080) | [-0.080, 0.233] |
| Role: security | 0.158 (0.142) | [-0.119, 0.436] | -0.001 (0.109) | [-0.214, 0.212] |
| Role: software development | -0.042 (0.026) | [-0.093, 0.009] | -0.017 (0.030) | [-0.075, 0.041] |
| AI exposure: current user | - | - | 0.211 (0.268) | [-0.314, 0.736] |
| AI exposure: no plan to use | - | - | 0.128 (0.269) | [-0.399, 0.654] |
| AI exposure: plans to use | - | - | 0.289 (0.271) | [-0.241, 0.820] |

Note. Model n was 22,757 in 2024 and 17,021 in 2025; $R^2$ was 0.327 and 0.446, respectively. Reference categories were job threat "not sure," age 18-24, and role "data and AI." The 2025 AI-exposure reference was missing/uncodable. AI exposure was constant among outcome-complete 2024 respondents and was omitted automatically. Country was used in prediction but not in the inferential RSA.

**Table 13.** Target-group signed calibration in 2025. Bias is prediction minus observed outcome. Entries are point estimates and 95% within-group respondent-bootstrap intervals; paired differences are TARS minus RSA.

| Type | Group | n | TARS bias [95% CI] | RSA bias [95% CI] | Paired Δbias [95% CI] |
|---|---|---|---|---|---|
| Role | Data and AI | 1,060 | -0.014 [-0.066, 0.046] | -0.012 [-0.065, 0.048] | -0.00188 [-0.00358, -0.00024] |
| Role | Infrastructure and operations | 654 | 0.093 [0.020, 0.163] | 0.097 [0.023, 0.167] | -0.00392 [-0.00581, -0.00201] |
| Role | Management and architecture | 2,036 | -0.058 [-0.099, -0.015] | -0.055 [-0.096, -0.012] | -0.00273 [-0.00377, -0.00163] |
| Role | Other technical | 385 | -0.000 [-0.084, 0.086] | 0.006 [-0.079, 0.092] | -0.00621 [-0.00902, -0.00333] |
| Role | Software development | 12,653 | -0.013 [-0.029, 0.003] | -0.009 [-0.025, 0.008] | -0.00427 [-0.00468, -0.00381] |
| Country | United States of America | 3,528 | -0.058 [-0.086, -0.026] | -0.051 [-0.079, -0.019] | -0.00685 [-0.00773, -0.00599] |
| Country | Germany | 1,512 | 0.057 [0.010, 0.100] | 0.062 [0.016, 0.105] | -0.00534 [-0.00656, -0.00392] |
| Country | India | 1,226 | -0.018 [-0.067, 0.032] | -0.020 [-0.070, 0.030] | 0.00213 [0.00093, 0.00339] |
| Country | United Kingdom | 921 | -0.016 [-0.073, 0.038] | -0.011 [-0.067, 0.043] | -0.00543 [-0.00708, -0.00372] |
| Country | France | 609 | 0.014 [-0.052, 0.089] | 0.020 [-0.046, 0.096] | -0.00630 [-0.00839, -0.00428] |
| Country | Canada | 602 | 0.015 [-0.057, 0.086] | 0.021 [-0.051, 0.092] | -0.00558 [-0.00779, -0.00352] |
| Country | Ukraine | 536 | -0.014 [-0.093, 0.066] | -0.013 [-0.092, 0.067] | -0.00076 [-0.00281, 0.00114] |
| Country | Other countries | 8,087 | -0.013 [-0.032, 0.008] | -0.010 [-0.029, 0.011] | -0.00312 [-0.00369, -0.00254] |

**Table 14.** Test using four sets of context variables. Coefficient changes are relative to the complete context set. Performance entries are paired TARS-minus-joint-model differences with 95% respondent-bootstrap intervals; negative RMSE and MAE differences favor TARS.

*Panel A. Change in Stage 1 coefficients*

| Context set | TARS maximum coefficient change | Joint maximum coefficient change | Joint relative $\ell_2$ change | Joint maximum change in Stage 1 prediction |
|---|---|---|---|---|
| Work and AI-use context | 0.0000 | 0.0202 | 4.04% | 0.0488 |
| Demographic context | 0.0000 | 0.0034 | 0.75% | 0.0105 |
| All context except geography | 0.0000 | 0.0191 | 3.83% | 0.0452 |
| Complete observed context | 0.0000 | 0.0000 | 0.00% | 0.0000 |

*Panel B. Paired performance on the 2025 test*

| Context family | Δ $R^2$ [95% CI] | ΔRMSE [95% CI] | ΔMAE [95% CI] | Δkappa [95% CI] |
|---|---|---|---|---|
| Work and AI-use context | 0.00036 [0.00028, 0.00043] | -0.00030 [-0.00036, -0.00023] | -0.00113 [-0.00119, -0.00106] | 0.00271 [0.00167, 0.00367] |
| Demographic context | 0.00149 [0.00109, 0.00182] | -0.00122 [-0.00150, -0.00089] | -0.00522 [-0.00553, -0.00490] | 0.01372 [0.01118, 0.01622] |
| All context except geography | 0.00038 [0.00029, 0.00047] | -0.00031 [-0.00038, -0.00023] | -0.00126 [-0.00134, -0.00119] | 0.00250 [0.00119, 0.00374] |
| Complete observed context | 0.00156 [0.00113, 0.00194] | -0.00128 [-0.00161, -0.00092] | -0.00577 [-0.00612, -0.00539] | 0.01028 [0.00825, 0.01254] |

**Table 15.** Stage 2 tuned for RMSE or MAE. Both TARS versions reuse the same Stage 1 coefficients; entries are 2025 point estimates and 95% respondent-bootstrap intervals. The one-stage references were independently tuned by validation MAE on the same source split.

| Model | Source selection metric | $\lambda_x$ or $\alpha$ | Maximum Stage 1 coefficient difference | $R^2$ [95% CI] | RMSE [95% CI] | MAE [95% CI] | Kappa [95% CI] |
|---|---|---|---|---|---|---|---|
| TARS-RMSE | RMSE | 100 | 0.0000 | 0.445 [0.435, 0.456] | 0.911 [0.902, 0.920] | 0.742 [0.734, 0.750] | 0.584 [0.575, 0.593] |
| TARS-MAE | MAE | 10 | 0.0000 | 0.445 [0.435, 0.456] | 0.911 [0.902, 0.920] | 0.741 [0.733, 0.749] | 0.584 [0.575, 0.593] |
| One-stage RSA-MAE | MAE | 10 | - | 0.443 [0.433, 0.454] | 0.913 [0.904, 0.922] | 0.748 [0.740, 0.755] | 0.574 [0.565, 0.583] |
| Trust main effects-MAE | MAE | 1 | - | 0.443 [0.432, 0.453] | 0.913 [0.905, 0.922] | 0.745 [0.737, 0.753] | 0.570 [0.561, 0.579] |

**Table 16.** Leave-one-group-out analysis. Each row removes the named group before 2024 tuning and fitting, then independently selects TARS-MAE and one-stage RSA-MAE on the remaining source data and evaluates the corresponding 2025 group. Entries are paired TARS-minus-RSA differences with 95% within-task respondent-bootstrap intervals; negative values favor TARS. Country buckets are defined from 2024 source frequencies.

| **Group type** | **Excluded 2024 group / 2025 test group** | **2025** n | **ΔRMSE [95% CI]** | **ΔMAE [95% CI]** |
|---|---|---|---|---|
| Role | Data and AI | 1,060 | -0.00484 [-0.00667, -0.00295] | -0.00793 [-0.00968, -0.00614] |
| Role | Infrastructure and operations | 654 | 0.00015 [-0.00176, 0.00199] | -0.00489 [-0.00673, -0.00294] |
| Role | Management and architecture | 2,036 | -0.00164 [-0.00267, -0.00057] | -0.00661 [-0.00763, -0.00557] |
| Role | Other technical | 385 | -0.00455 [-0.00709, -0.00206] | -0.01017 [-0.01247, -0.00777] |
| Role | Software development | 12,653 | 0.00024 [-0.00004, 0.00051] | -0.00270 [-0.00296, -0.00244] |
| Country | Canada | 602 | -0.00401 [-0.00652, -0.00160] | -0.00982 [-0.01225, -0.00721] |
| Country | France | 609 | -0.00704 [-0.01060, -0.00381] | -0.01635 [-0.02105, -0.01165] |
| Country | Germany | 1,512 | -0.00451 [-0.00594, -0.00307] | -0.00818 [-0.00962, -0.00669] |
| Country | India | 1,226 | -0.00229 [-0.00311, -0.00148] | -0.00415 [-0.00491, -0.00338] |
| Country | Other countries | 8,087 | -0.00242 [-0.00301, -0.00182] | -0.00667 [-0.00724, -0.00612] |
| Country | Ukraine | 536 | -0.00161 [-0.00445, 0.00127] | -0.00610 [-0.00984, -0.00210] |
| Country | United Kingdom | 921 | -0.00316 [-0.00503, -0.00149] | -0.00920 [-0.01100, -0.00745] |
| Country | United States of America | 3,528 | -0.00335 [-0.00428, -0.00243] | -0.00920 [-0.01011, -0.00823] |
